# Molecular Subtypes of Pancreatic Neuroendocrine Tumors Mutated in *MEN1/DAXX/ATRX* Explain Biological Variability

**DOI:** 10.1007/s12022-025-09889-6

**Published:** 2025-11-10

**Authors:** Simona Avanthay, Annunziata Di Domenico, Philipp Kirchner, Konstantin Bräutigam, Aziz Chouchane, Renaud Maire, Christina Thirlwell, Corina Kim-Fuchs, Aurel Perren, Ilaria Marinoni

**Affiliations:** 1 Institute of Tissue Medicine and Pathology, University of Bern, Bern, 3008 Switzerland; 2 Graduate School for Cellular and Biomedical Sciences, University of Bern, Bern, Switzerland; 3 Bristol Medical School, University of Bristol, Bristol, England UK; 4 Department of Visceral Surgery and Medicine, University Hospital Bern, University of Bern, Bern, Switzerland

**Keywords:** Epigenome, Transcriptome, Pancreas, Neuroendocrine, Tumor

## Abstract

About 40% of non-functioning (NF) Pancreatic Neuroendocrine Tumors (PanNETs) harbour mutations in *MEN1*, often co-occurring with *DAXX/ATRX.* While the ADM group (*MEN1* and *DAXX/ATRX* co-mutated) exhibits homogeneous genetic and epigenetic features and a consistent risk of relapse, it shows considerable variability in treatment response, suggesting an underlying molecular diversity. In this study we aimed to elucidate the molecular mechanisms underlying this heterogeneity of ADM PanNETs by integrating transcriptomic (*n* = 36) and DNA methylation (*n* = 93) data. First, DNA methylation discriminates ADM-PanNET from PanNET mutated only in *MEN1* (α-like), revealing enhancer, peri-centromeric, and telomeric methylation changes associated with alternative lengthening of telomeres and increased chromosomal instability. Transcriptomic analysis further revealed three distinct ADM subtypes: ADM hypoxic, ADM NST (No Special Type), and ADM immunosuppressive. The ADM hypoxic subtype is characterized by strong hypoxia signature, likely regulated epigenetically. The ADM NST subtype appears to be primarily driven by epigenetic changes that promote proliferation. Notably, the ADM immunosuppressive subtype, although significantly smaller (< 2.5 cm, *p* = 0.023), exhibits strong immune and metastasis-like signatures, suggesting a uniquely aggressive biology despite its reduced size. By defining these three novel ADM subtypes, our study provides a refined framework for understanding PanNET heterogeneity. This classification underscores potential diagnostic markers and highlights distinct biological vulnerabilities that may inform the development of subtype-tailored therapeutic strategies.

**Supplementary Information:**

The online version contains supplementary material available at 10.1007/s12022-025-09889-6.

## Introduction

Although direct evidence is still lacking, the most probable origin of Pancreatic Neuroendocrine Tumors (PanNETs) is in the islets of Langerhans, which include 5 different cell types: α, β, γ, δ and ε-cells. PanNETs are heterogenous, as they seem to arise from different types of cells and have different mutational backgrounds. They can be functioning (associated with clinical symptoms of hormone excess), or non-functioning (hormone production not causing overt clinical symptoms) [1]. Non-Functioning PanNETs (NF-PanNETs) seem to mainly originate from α-cells and frequently develop metastases [2]. Compared to other solid tumors, the background mutation rate is very low [3]. NF-PanNETs with mutations in *MEN1*, *DAXX* and *ATRX* occur in almost 40% of the patients, making them the most frequent genomic PanNET type [4, 5]. Mutations in *DAXX* and *ATRX* are generally mutually exclusive and correlate with subsequent loss of nuclear protein expression [4–6]. DAXX and ATRX function as chromatin remodelers and are responsible for both DNA methylation and histone H3.3 deposition in specific heterochromatic regions [7–9]. Loss of nuclear DAXX and ATRX expression in PanNETs is associated with global DNA hypomethylation, enhanced chromosomal instability and alternative lengthening of telomeres (ALT), a mechanism responsible for telomere elongation independent from telomerase activation [6, 10, 11]. PanNETs with DAXX/ATRX loss of function are a heterogeneous group and tumors harboring these mutations show variable clinical courses without a common response to specific treatments [12]. Although the loss of DAXX/ATRX function is strongly predictive of liver metastasis formation, its role in later stages of progression remains unclear, with other mechanisms appearing to take over [12, 13]. For instance, Alvarez and colleagues show mutation-independent oncogenic reliance on early neuroendocrine lineage factors, EMT drivers, and immunomodulatory factors during metastatic progression [14]. Similarly, another study identified specific PanNET subtypes based on their immunogenic profiles irrespective of their mutational background [15]. Finally, in rare cases, additional mutations in *TP53* and *RB1* can drive progression towards more aggressive, high-grade phenotype [16, 17].

Understanding this observed PanNET heterogeneity is crucial to improve treatment selection, prognostication and follow up protocols. To date, integrated genomic studies have described clinically and biologically distinct PanNET subgroups [2, 5, 15, 18]. Transcriptome studies have identified at least three PanNET subtypes including one with potential clinical relevance, characterized by a metastasis like signature, in conjunction with increased tumor size, hypoxia, necroptosis and enhanced immune gene expression [5, 15, 18]. Yet, this stratification has limited clinical application. Epigenetic analyses including DNA methylation profiles distinguish PanNETs with different risks of relapse, mutational drivers as well as different cells of origin [2, 19, 20]. We have previously described three main tumor subtypes through DNA methylation analysis: β-like tumors, with an epigenetic signature resembling normal β-cells, α-like tumors resembling normal α-cells, and an intermediate group of tumors with an epigenetic profile retaining a partial α-cell- signature [2]. The group of α-like PanNETs were clinically non-functioning, small, indolent, enriched in only *MEN1* mutation and chromosomally stable.

Intermediate PanNETs were larger, carried a high risk of relapse, and were enriched for tumors harbouring mutations in both *MEN1* and *DAXX/ATRX* (ADM tumors). They also exhibited high chromosomal instability and lacked functional syndromes. Both α-like and ADM tumors show features of endocrine pancreatic α-cells including ARX expression, which is the transcription factor driving α-lineage differentiation; hence we postulated that these PanNETs may originate from these cells [2]. Based on these findings we have hypothesized a possible stepwise progression for NF-PanNETs developing from α-cells into ADM tumors [2]. Nevertheless, as mentioned above, the ADM tumor group remains highly heterogenous, and different mechanisms may drive further progression.

Interestingly, while epigenetic groups correlate with underlying somatic mutations, the groups defined by transcriptome analysis do not [5, 15]. Transcriptome profiles clearly identify a group of advanced NF-PanNET with metabolic changes and activated hypoxia signaling, while this aggressive PanNET subtype is not observed in epigenetic studies [2]. Conversely, transcriptome analysis does not discriminate small indolent PanNETs with low risk of relapse [15]. Therefore, multi-omic analyses integrating different modalities are crucial to obtain a more comprehensive molecular classification of PanNETs. The key aim of this study is to molecularly dissect the heterogeneity of ADM tumors and to identify possible mechanisms of progression and vulnerabilities within this group. To this end, we integrated genomic, transcriptomic, and DNA methylation data for a cohort of PanNETs with loss of protein expression of Menin, DAXX, or ATRX. We could describe three distinct subtypes in the ADM group, all showing increased proliferation (tumor grade) compared to α-like: one characterized by hypoxia signaling, one enriched in immune components, and one with no specific signature except an increased proliferation rate. While ADM tumors have a similar clinical course the ADM immunosuppressive were smaller in size.

## Methods

### Cohorts and Samples

Data included three independent cohorts of well-differentiated PanNETs (in total *n* = 102, clinic-pathological and molecular data in Supplementary Table 1, Supplementary Fig. 1A and 1B): Di Domenico et al. [2], Chan et al. [20], Scarpa et al. [5]), 8 additional primary tumors and 9 matched metastases from the Institute of Tissue Medicine and Pathology, University of Bern [2]. Since DNA mutations in *MEN1*, *DAXX*, and *ATRX* are strongly correlated with their nuclear protein expression, loss or retention of Menin, DAXX, and ATRX were inferred from immunohistochemistry or obtained from DNA sequencing when immunohistochemistry was unavailable or uncertain [6, 21, 22]. Only cases with loss of Menin, DAXX, or ATRX were included.

Supplementary Fig. 1A provides a flow-chart of the samples used for each analysis.

### Nucleic Acid Extraction

Genomic DNA was extracted from formalin-fixed paraffin-embedded tissues using the QIAamp DNA minikit (Qiagen) according to manufacturer’s recommendation. Serial sections were macrodissected using a razor blade upon histological evaluation (5 × 6 μm), tumor cell content was assessed on corresponding H&E-stained slides by a trained pathologist (A.P.). Only samples with at least 70% tumor cell content were included. DNA quality was assessed using the Illumina FFPE QC Kit.

For extraction of total RNA from frozen tissue, the Single Cell RNA Purification Kit (Norgen Biotek, #51800) was used. RNA quantity and purity were quantified with the Qubit DNA/RNA HS detection kit (Thermo Fisher Scientific, #Q32852). All samples satisfy a minimum RNA quality value (RQN) of 5.

### RNAseq Data Analysis

For library preparation, 1 ug of high-quality RNA was used in the Illumina TruSeq RNA library prep kit according to the manufacturer’s recommendations. Libraries were sequenced as paired end 50 bp reads on a NovaSeq 6000 (Illumina) platform at approximately 20 M reads/sample. Adapter sequences and homopolymer reads were trimmed from reads with a minimum length of 40 bp after trimming using cutadapt v3.4.1. Read quality was checked before and after trimming using FastQC v0.9.11. High confidence ribosomal reads were removed with ribodetector v0.2.7 using default settings [23, 24]. Filtered reads were mapped to the human reference genome GRCh38 with the GENCODE v38 gene model using STAR v2.7.3a. Mapped reads were assigned to genes using subread feature counts v2.0.1 [25, 26], discarding reads overlapping more than one gene annotation.

Further FASTQ PanNET data from Chan et al. [20] were downloaded from Gene Expression Omnibus (GEO) (http://www.ncbi.nlm.nih.gov/geo/, accession number: GSE118014). Raw FASTQ files were probed for sequencing quality control using FastQC and low-quality samples were excluded. Sequencing reads were mapped to the human reference genome (ENSEMBL hg19) with the GENCODE v19 gene model using STAR 2.7.3a. Mapped reads were quantified using RSEM 1.3.0 with default parameters [27].

For additional G1/G2 PanNET cases from Scarpa et al. [5], RSEM count matrices were downloaded from the ICGC Data Portal (https://icgc.org/, PAEN-AU project). We merged the raw counts of the three datasets and batch corrected them using the ComBat_seq algorithm from the sva (v3.50.0) R package [28]. Only raw counts and batch factor were included as inputs, default parameters were kept, and the group variable was not specified. Filtration for low count genes was performed to have a minimum of 2 samples with at least 5 reads per gene. Further normalization and differential gene expression analysis were performed using the DESeq2 (v1.42.1) R package [29], following the default settings. Differential expression *p* values were adjusted for multiple testing using the Benjamini-Hochberg method. Effect size shrinkage was applied to the estimated log fold change using the apeglm algorithm with default parameters [30]. For other downstream analyses (visualization, clustering, correlation) we used VST transformed data.

### DNA Methylation Data Analysis

Genomic DNA was prepared for methylation analysis as described previously [2]. DNA was processed on the Infinium Methylation HM450, MethylationEPIC or MethylationEPICv2 array (Illumina). IDAT files containing the raw data from Infinium HM450, EPIC, and EPICv2 arrays were loaded, filtered and pre-processed as implemented in Sesame (1.24.0) pipeline (qualityMask, inferInfiniumIChannel, dyeBiasNL, pOOBAH, noob) [31]. The out-of-band *p* value (pOOBAH) threshold was relaxed to 0.2 and only probes failing this threshold in more than 10% of samples were excluded. Probes with systematic problems collected in the static mask were excluded and only probes located on autosomal chromosomes were retained. LiftOver function from Sesame (1.24.0) package was used to reach consensus between probe names on EPICv2 and the other two microarray types. Subsequently, the β values of probes present in all three microarray types were combined. Differentially methylated sites between the described PanNET sub-clusters were identified via linear model fitting from limma (3.62.2) R package using β values [32]. Differential methylation *p* values were adjusted for multiple testing using the Benjamini-Hochberg method.

### Correlation Between Probe Methylation and Gene Expression

To select genes possibly regulated via DNA methylation, Spearman correlation was calculated between DNA logit transformed methylation β values (M values) and VST transformed counts for samples that had both methylome and transcriptome data available. Only differentially expressed genes were taken into consideration (|logFC|>1, adjusted *p*-value < 0.05). Correlation *p* values were adjusted for multiple testing using the Benjamini-Hochberg method. The selection criteria for candidate probe-genes associations were Rho value < −0.6 and adjusted *p*-value < 0.05.

### Consensus Clustering

Consensus clustering was performed using 2000 most variable probes (β values) or genes. The same probes were used for analysis including primary tumors and matching metastases. In all analyses we used the same parameters (ConsensusClusterPlus (v1.66.0) [33]; Pearson correlation, maxK = 20, reps = 1000, pItem = 0.8, pFeature = 1). To evaluate consensus and cluster confidence, we used the relative change in area under the consensus cumulative distribution function (CDF) as implemented in Consensus Cluster Plus [33]. Samples were clustered according to the hierarchical clustering algorithm, ward.D2 method was used for inner linkage and average method was used for the final linkage.

### Statistics, and Graphical Representation

Statistical analysis and graphical representation were carried out in R version 4.3.3 [34]. Packages used for data pre-processing and comparative analysis with respective parameters are mentioned in the previous chapters. Highly variable probes or genes were selected using the median absolute deviation (MAD). Gene Set Enrichment and overrepresentation analysis of MSigDB 7.1.1 Hallmark pathways was performed within the clusterProfiler (v4.10.1) R package [35]. For disease free survival analysis, the survival (v3.5–8) and survminer (v0.4.9) packages were used [36, 37]. Overrepresentation analysis (one sided Fisher’s exact test) was used to identify chromatin regions enriched in methylation changes. Metastasis-like primary 1 (MLP1) [15] and Group 3 [5] score were determined as the sum of ranks of signature genes using colRanks from MatrixGenerics package (v1.14.0) [5, 15, 38]. Pearson’s Chi-squared test with simulated *p*-value was used to determine association of ADM subtypes and tumor size (smaller or larger than 2.5 cm) and perineural or vascular space invasion.

### Immunohistochemistry and Telomeric-Fluorescence in Situ Hybridization (Telo-FISH)

Immunohistochemistry was performed using a BOND RX Fully Automated Research Stainer on whole tissue slides of patients with available DNA methylation profile. Slides stained for MCT4 (1:50, anti-MCT4, monoclonal mouse D1, sc376140; Santa Cruz, Heidelberg) were pretreated in TRIS-EDTA (pH 9, BOND Epitope Retrieval Solution 2) at 95 °C for 30 min. MCT4 protein expression was scored as follows: negative (< 20% expression); homogenous positive (> 80% expression); heterogeneous (areas of positive and negative expression in association with microvessels, 20% to 80% expression). Slides for Menin (1:800, anti-Menin, A300-150 A, Bethyl Laboratories) were pretreated with HIER citrate buffer for 32 min at 100 °C or 30 min at 95 °C, respectively. Staining of DAXX and ATRX and Telo-FISH were performed as described in Di Domenico et al., 2020 [2]. In brief, telomere FISH deparaffinized sections were hybridized with a peptide nucleic acid (PNA) probe (telC-Alexa488; Panagene, Daejeon, Korea) diluted (1:10) in 70% formamide, 10 nmol/L Tris, pH 7.5. FISH was evaluated using an Olympus VS 110 Fluorescent Scanner (Olympus, Volkestwil, Switzerland). ALT-positive sample showed a mixture of very bright large and very faint small telomeric signals, a hallmark of high heterogeneity in the size of telomeric sequences.

### Copy-Number Alteration (CNA) Analysis

Copy number alteration (CNA) data were obtained from previously published studies, in which genome-wide CNAs were inferred from HM450 methylation array data using the conumee R package [2, 5]. CNA profiles were manually thresholded based on FISH validation, as described in [2]. We did not perform additional CNA analysis.

## Results

### Integrated Methylome Analysis Identifies Two PanNET Subtypes with Loss of ATRX/DAXX and Menin

**Fig. 1:**
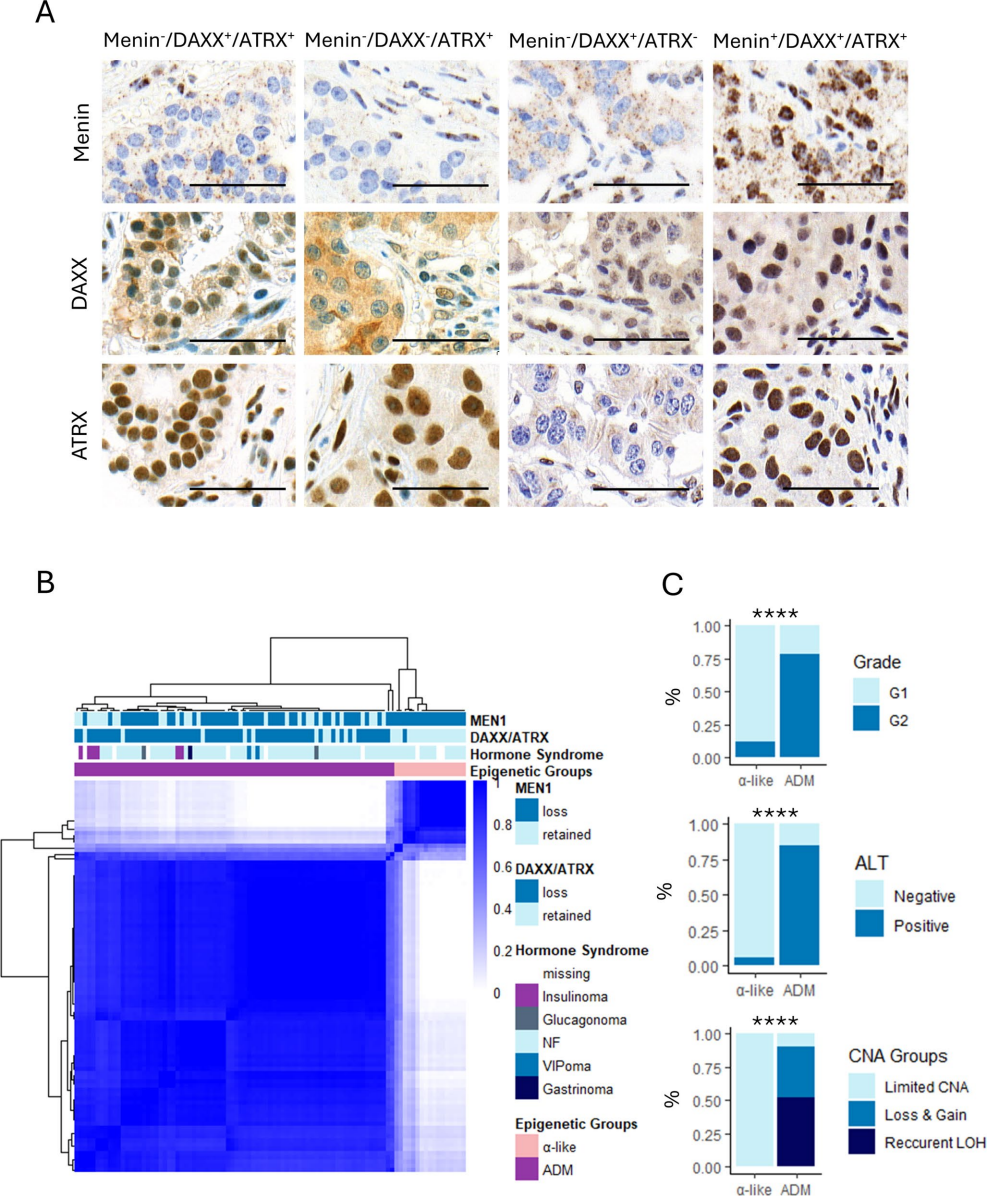
PanNETs with loss of DAXX/ATRX and Menin can be divided into 2 groups according to DNA methylation. **A** Representative immunohistochemistry of loss (^−^) and retention (^+^) of Menin, DAXX, and ATRX. Each column represents one sample; each row corresponds to immunohistochemistry staining of the reported protein. Scale bars in the right bottom represent 50 μm. **B** Consensus clustering of the 93 PanNETs with loss of DAXX/ATRX and/or Menin loss according to the top 2000 most variably methylated probes (selected by MAD) shows segregation of α-like and ADM PanNETs. Consensus cluster correlation is indicated in the heatmap with darker colors representing stronger correlation. Each column represents one sample. Menin and DAXX/ATRX status, hormone syndrome, and epigenetic subtypes are indicated according to the reported colors. **C** ADM PanNETs are strongly associated with higher grade, alternative lengthening of telomeres (ALT), and copy number alteration (CNA) (Pearson’s Chi-squared test, ****p-value < 0.0001). LOH = loss of heterozygosity

To dissect the heterogeneity of the ADM PanNET subtype, we performed DNA methylation analysis of samples with loss of nuclear expression of at least one of the α-cell–associated proteins: Menin, DAXX, or ATRX (*n* = 93, Fig. 1A).

To recapitulate the α-like and ADM PanNET epigenetic subtypes, we performed consensus clustering using the 2000 most variably methylated probes [2, 20]. When employing two clusters, DNA methylation profiles clearly distinguish subtypes which are strongly associated with loss of DAXX/ATRX expression, in accordance with previous findings [2]. Therefore, the cluster enriched in tumors with loss of Menin, but not DAXX/ATRX, was labelled α-like, while the cluster enriched in samples with loss of Menin and DAXX/ATRX was referred to as ADM (Fig. 1B). Importantly, ADM PanNETs were associated with higher grade (Pearson’s Chi-squared test, *p*.value = 7.0 × 10^−6^), activation of alternative lengthening of telomeres (ALT, Pearson’s Chi-squared test, *p*.value = 4.0 × 10^−8^), and chromosomal instability (Pearson’s Chi-squared test, *p*.value = 2.3 × 10^−8^), indicating more aggressive phenotype (Fig. 1C). The vast majority of included samples were non-functioning (*n* = 72), followed by insulinomas (*n* = 6), VIPomas (*n* = 2), and one gastrinoma. Out of the six insulinomas, four were positive for ARX and negative for PDX1, one was negative for ARX and positive for PDX1, and for one sample the immunohistochemistry was not available.

### Hypomethylation of Telomeric and Peri-Centromeric Regions is Associated with Increased Chromosomal Instability in ADM PanNETs

To investigate which specific epigenetic changes occur between α-like and ADM PanNETs, we performed differential methylation analysis, which revealed 6316 CpG probes (DMPs, p.adj < 0.001, |Δβ| >0.2, Supplementary Table 2).

**Fig. 2:**
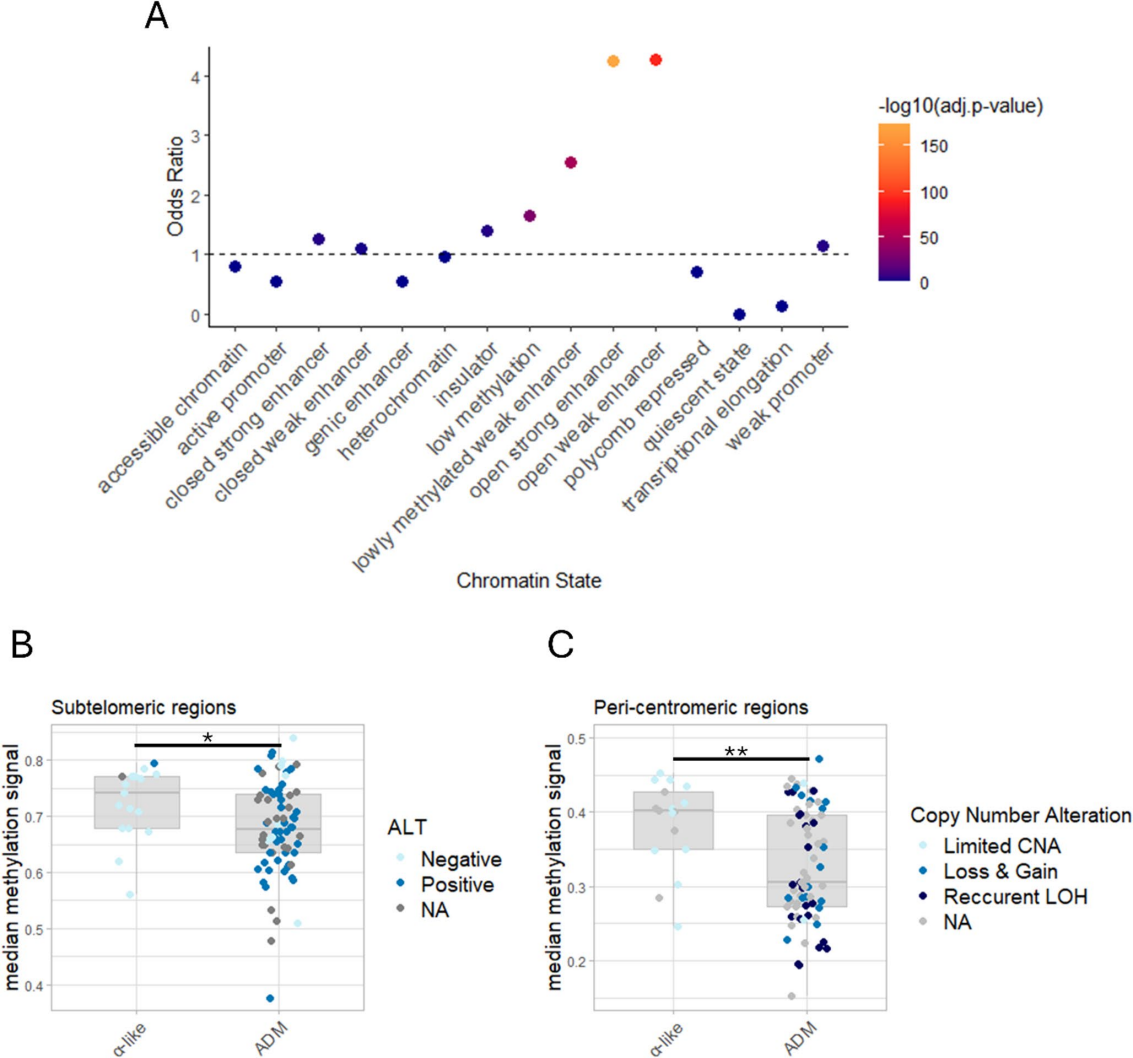
Characteristic DNA methylation differences between the three NF-PanNET subgroups. **A** Graphical representation of DMP distribution across chromatin states based on over-representation analysis (one-tailed Fisher’s exact test). Odds ratio > 1 (above dotted line) indicates region enriched in the DMPs. Adjusted *p*-values for each region are -log_10_ transformed and indicated according to the color key. **B** and **C** Median methylation signal of subtelomeric (B) or peri-centromeric (C) regions are significantly lower in ADM PanNETs (t-test, *p-value < 0.05, **p-value < 0.01). Each dot represents one sample. Presence of alternative lengthening of telomeres (ALT) or copy number alteration (CNA) is indicated according to color legend. NA designates samples for which ALT or CNA status was not available. LOH- loss of heterozygosity

We interrogated the location of these probes in the islet-specific genomic feature atlas defined by Thurner et al. [39]. Over-representation analysis revealed enrichment in enhancers and lowly-methylated regions, suggesting that the changes between α-like and ADM tumors affect mainly non-coding regions (Fig. 2A).

Hypermethylation of heterochromatin, especially at subtelomeric and pericentromeric regions, is crucial for maintenance of chromosomal stability [40]. To investigate the association between epigenetic changes, ALT and copy number alteration (CNA) in PanNETs we assessed the median methylation levels of sub-telomeric and peri-centromeric regions. Both regions showed higher median methylation in the α-like compared to the ADM PanNETs (Fig. 2B and C), linking the loss of methylation in structural regions with ALT activation and genomic instability. Methylation levels of specific chromosomes on sub-telomeric and peri-centromeric regions are depicted in Supplementary Fig. 2A and Supplementary Fig. 2B, respectively. Notably, chromosomes 3,5,7,9,11,14,17,19, and 20 are hypomethylated in subtelomeric regions, while chromosomes 1,2,6,7,11,12,13,14,17, and 22 are hypomethylated in peri-centromeric regions.

### Progression from α-like to ADM

**Fig. 3:**
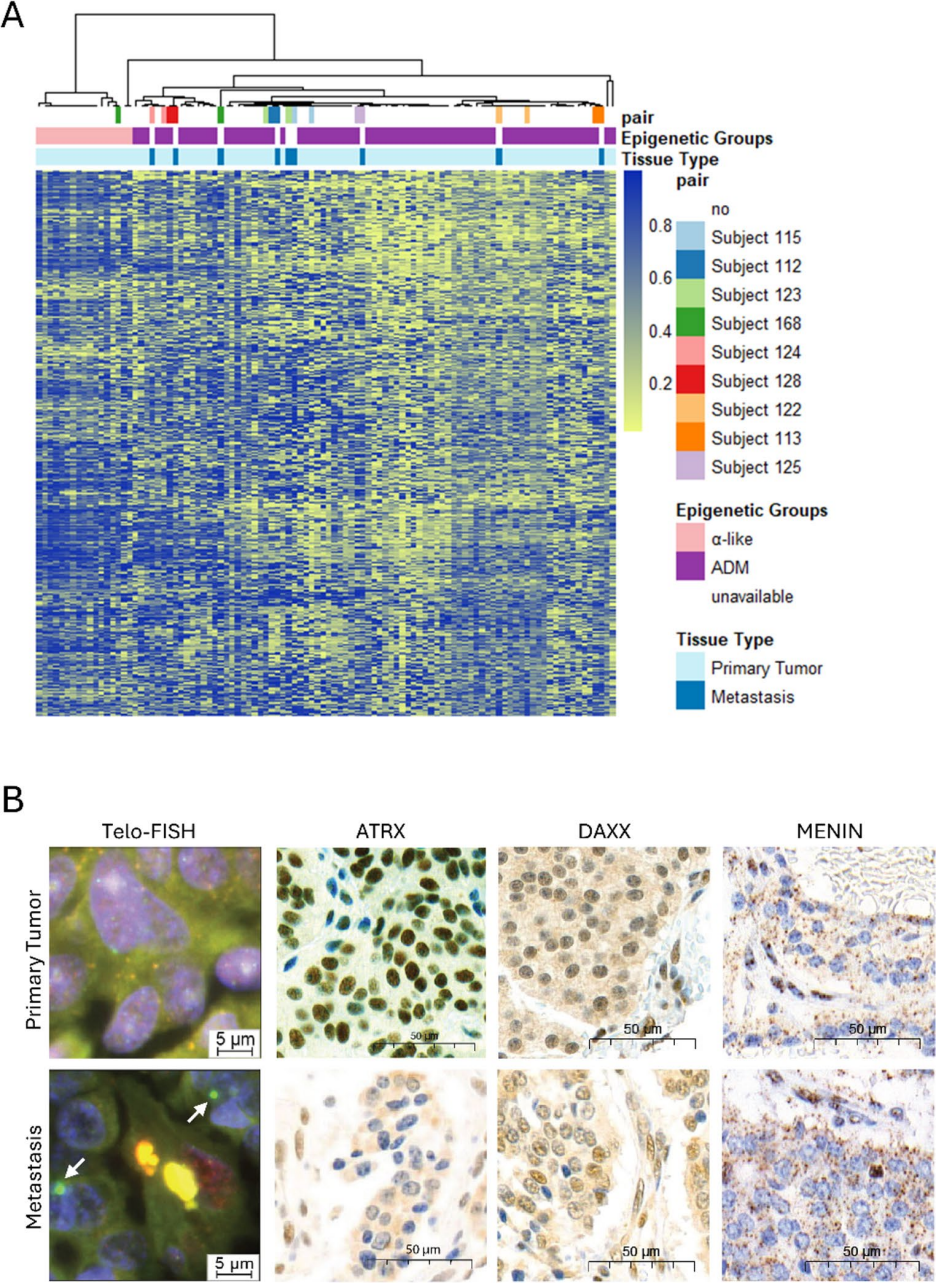
Alpha-line metastatic NF-PanNET shows epigenetic progression. **A** Consensus clustering of 93 primary PanNETs and 9 matching metastases according to the 2000 most variable probes from Fig. 1B. Consensus clustering dendogram was obtained for k = 2. The yellow to blue color scale indicates the DNA methylation value (β value, 0 = unmethylated, 1 = methylated) for each probe (in rows). Each column represents one sample. Matching patient samples and tissue type are indicated according to the color legend. Subject 168 (in green) shows progression from α-like primary tumor into ADM metastasis. **B** Telo-FISH shows activation of alternative lengthening of telomeres in metastasis (white arrows), but not in primary tumor. Immunohistochemistry for ATRX is preserved in primary tumor and lost in metastasis. Both primary tumor and metastasis show Menin loss and DAXX retention

Epigenetic and genetic evidence suggests that ADM tumors could develop from α-like tumors upon DAXX/ATRX loss of function [2].To assess this possibility, we conducted consensus clustering including an additional set of nine matching metastases (Supplementary Table 1, Supplementary Fig. 1A and 1B). One sample pair was treated with neoadjuvant chemotherapy (streptotozin-doxorubicin) before resection of both primary tumor and metastasis (Subject 123). All other pairs were treatment naïve. Consensus clustering used the same probes that distinguished α-like and ADM PanNETs also targeting two clusters. Interestingly, one sample pair segregated between α-like and ADM tumors (subject 168, Fig. 3A). Notably, the primary tumor clustering with α-lineage PanNETs showed ATRX retention, while the matching metastasis clustered with the ADM tumors and was ATRX negative (Fig. 3A and B). Furthermore, ALT was negative in the primary tumor, but positive in the metastasis (Fig. 3D and E, respectively). These results provide evidence for progression from α-like to ADM NF-PanNET in one patient.

### Transcriptomic Analysis Distinguishes 4 Subtypes of ADM PanNET

Next, we performed gene expression analysis for a subgroup of 36 samples for which we had RNA-seq data available in addition to the DNA-methylation data (Supplementary Fig. 1, Supplementary Table 1).

Consensus clustering of these samples using the 2000 most variably expressed genes (selected by MAD) did not recapitulate the epigenetic α-like trait. The α-like PanNETs did not form a separate cluster (Supplementary Fig. 3A), but were intermixed in the ADM PanNETs, suggesting that transcriptomic profiling alone is insufficient to distinguish between the two entities. Since the primary focus of this study was to investigate heterogeneity within ADM PanNETs, we excluded the five α-like PanNETs from further analysis, resulting in a final set of 31 ADM samples.

**Fig. 4:**
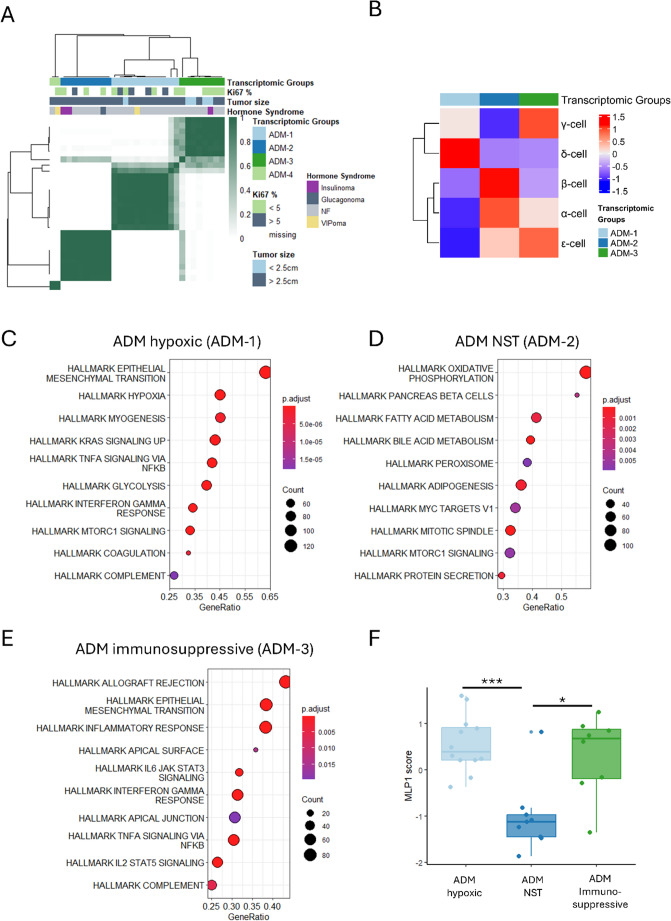
ADM PanNETs are transcriptionally heterogeneous. **A** Consensus clustering of ADM PanNETs based on 2000 most variably expressed genes (selected by MAD). Each column represents a sample. Darker colors in the heatmap represent stronger cluster correlation. Hormone syndrome and transcriptomic subtypes are indicated according to the color key (NF- non-functioning). **B** Heatmap of scaled mean expression (in rows) of transcription factors linked with α-, β-, γ-, δ-, and ε-cell per consensus cluster (in columns). Transcriptomic subtypes are indicated by color. Blue and red color in the heatmap represents low and high expression, respectively. **C-E** Gene set enrichment analysis based on DEGs identified for each group. Top 10 up-regulated hallmark pathways (indicated on the left) are depicted. Adjusted *p*-value colored by the color key. **F** MLP1 gene signature score (y axis) per group (x axis). Boxplots are colored according to the transcriptomic groups

Consensus clustering on the 2000 most variably expressed genes (selected by MAD) confirmed transcriptomic heterogeneity of ADM PanNETs (Fig. 4A). Good cluster stability could be obtained with 4 clusters (ADM-1 to ADM-4). Higher resolutions further increased stability but also resulted in the creation of additional small clusters (Supplementary Fig. 3B and C). Of note, the cluster 4 (ADM-4) forms very early (k = 2) and remains stable until k = 10, underscoring its specific transcriptomic profile (Supplementary Fig. 3C). However, due to the small sample size (*n* = 2) it had to be excluded from further analysis. Our analysis did not reveal any differences in risk of metastasis formation, relapse, and perineural or vascular space invasion between the four ADM groups (Supplementary Fig. 3D-G). However, the ADM-3 PanNETs were significantly enriched in tumors smaller than 2.5 cm (Fig. 4A, chi-squared test with simulated *p* value, *p* value = 0.023, median = 2.4 cm), and the ADM-1 seem to have higher Ki67 albeit not significant likely due to the small sample size (Fig. 4A, Supplementary Fig. 3H). While the majority of included PanNETs are non-functioning, we have also three insulinomas (two in ADM-2, one in ADM-3), two VIPomas (one clustering with ADM-4, one with ADM-1), and one glucagonoma in ADM-1. Of note, two insulinomas and the glucagonoma were ARX positive and PDX1 negative, as assessed by immunohistochemistry. The immunohistochemistry was not available for the VIPomas and one insulinoma.

Investigation of islet-cell specific transcription factors (TFs) defined by Muraro et al. revealed differences among the groups [41]. The Kruskal–Wallis test confirmed significant differences in the expression of transcription factors associated with α- (adjusted *p*.value = 0.049), β- (*p*.value = 0.033), and δ- (*p* = 0.0241) cells across the ADM groups. Specifically, ADM-2 exhibited the highest expression of transcription factors linked to α- and β-cells, ADM-1 was enriched for δ-cell transcription factors, and ADM-3 showed elevated expression of those associated with γ- and ε-cells. (Fig. 4B).

In order to characterize the specific ADM subtypes, we performed differential gene expression analysis between each group and the rest of the cohort. We identified similar number of differentially expressed genes (DEGs, |log2FC|>1, adjusted *p*-value < 0.05) for ADM-1 (870, Supplementary Table 3) and ADM-2 (899, Supplementary Table 4). ADM- 3 rendered the lowest number of DEGs (498, Supplementary Table 5).

Interrogation of Hallmark pathways enriched in individual clusters revealed characteristic metabolic changes in ADM-1, notably up-regulation of hypoxia and glycolysis (Fig. 4C). Therefore, we refer to ADM-1 cluster as ADM hypoxic. Other up-regulated pathways were involved in cell cycle (E2F targets, G2M checkpoint), epithelial to mesenchymal transition (EMT), mTOR, KRAS or TNFA signaling, complement and coagulation (Fig. 4C). ADM- 2 showed different metabolic changes (oxidative phosphorylation, fatty acid metabolism, and adipogenesis). Of note, this cluster comprises three functioning tumors, including two insulinomas, which could explain the enrichment in pancreatic β-cells and protein secretion pathways (Fig. 4D) and increased expression of β-cell TFs (Fig. 4B). Other pathways encompass bile acid metabolism, peroxisome, and mitotic spindle (Fig. 4D). Since this cluster did not present any specific characteristics, we named it ADM NST (no special type). ADM-3 showed enrichment mainly in inflammation and immune-related pathways (allograft rejection, inflammatory and interferon γ response, and IL2 and IL6 signaling) (Fig. 4E). Therefore, we labelled it ADM immunosuppressive.

Importantly, Young and colleagues previously characterized MLP1 subtype with strong hypoxic and immunosuppressive transcriptional phenotype [15]. In accordance, ADM hypoxic and ADM immunosuppressive show higher MLP1 transcriptomic signature compared to the ADM NST (Fig. 4F).

### ADM Hypoxic Signalling is Epigenetically Driven

Next, we sought to understand whether the identified transcriptomic changes could be driven by DNA methylation of promoters. Therefore, we investigated the correlation between methylation of CpG sites and corresponding differentially expressed genes (adjusted *p*-value < 0.05, |log2FC| >1). The available DNA methylation data set was limited to probes mapping to annotated active or weak promoter regions [39]. Only DEGs that showed negative correlation (adjusted *p*-value < 0.05, Rho < −0.6) with at least one CpG site in their promoter region were selected.

**Fig. 5:**
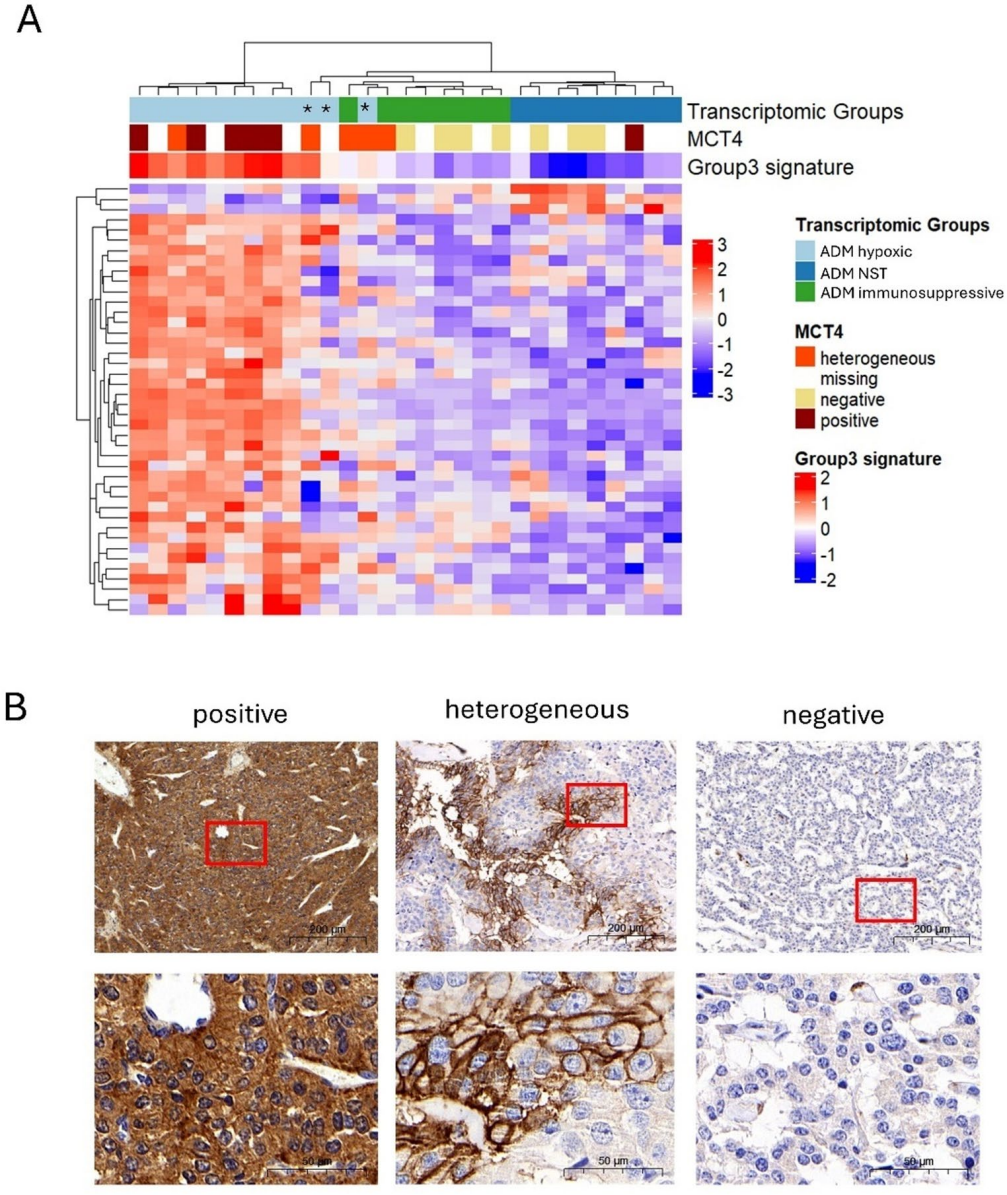
Integration of methylome and transcriptome reveals changes in metabolic genes. **A** Hierarchical clustering of ADM PanNETs according to 42 DEGs with anti-correlated promoter DNA methylation and gene expression. Each column represents a sample, each row represents a gene. The scaled hypoxic group 3 score defined by Scarpa et al. [5] is indicated by color key (blue- low, red-high). PanNET subgroups for each sample are reported. Blue and red in the main matrix corresponds to gene expression as indicated by color key. The asterisks indicate three ADM-hypoxic samples that cluster separately. **B** Immunohistochemistry of MCT4 on representative samples. Each column shows one of the distinct staining patterns used in scoring. The red square delimitates area that is magnified below

With this approach we identified 39 up-regulated and 3 down-regulated DEGs inversely correlated with methylation of corresponding promoters in ADM hypoxic tumors (Fig. 5A, Supplementary Table 6). Importantly, many of the identified genes were involved in hypoxia response (ex. *MCT4/SLC16A3*,* CA9*) including lactate metabolism (ex. *LDHA*,* LDHB*) and glycolysis (ex. *P4HA2*,* PLOD1*), indicating that the metabolic phenotype may be epigenetically driven. Other correlated genes, such as *VIM*,* PDLIM4*, and *IGFBP4* are involved in epithelial to mesenchymal transition. Immunohistochemistry confirmed expression of hypoxic markers, such as SLC16A3/MCT4 and CA9 on protein level (Fig. 5B). Cluster analysis based on the identified DEGs-promoter association showed separation of three samples (Subject 36, Subject 49, and Subject 10, indicated by an asterisk in Fig. 5A) from the ADM hypoxic group (Fig. 5A). This could be explained by the heterogeneous MCT4 in two out of three samples, and for one sample the MCT4 was not available. Possibly these PanNET are truly hypoxic with a micro-vessel gradient. The hypoxic signature defined by Scarpa et al. in two of these samples was the lowest among ADM hypoxic subtype (Fig. 5A) [5]. The observed correlation between epigenetic and transcriptomic profiles of ADM hypoxic PanNETs prompted us to investigate whether they could be also discriminated on DNA methylation level. Interestingly, a principal component analysis (PCA) based on 2000 most variably methylated probes showed close clustering of MCT4 homogeneously positive samples, confirming that they also share a specific epigenetic signature (number of samples with available MCT4 immunohistochemistry = 40; Supplementary Fig. 4).

Finally, analysis of DEG-probes correlation rendered remarkably fewer genes for the rest of ADM groups. Indeed, only five up-regulated DEGs were correlated with promoter methylation in ADM-NST (*NR0B2*,* CDKN1C*,* C2CD4B*,* FERMT1*,* CASR*) and two in ADM-immunosuppressive (*GFI1* and *HSPA1A)* (Supplementary Tables 7 and 8). Resolving the interplay of epigenetics and gene expression in these groups will require additional studies.

## Discussion

Multi-omic analysis of PanNET DNA methylation and gene expression revealed a two-level hierarchy. First, DNA methylation was able to separate samples based on their mutational profiles, chromosomal instability and ALT phenotype, as well as epigenetic similarity to α-cells. ADM samples showed increased epigenetic dysregulation compared to more differentiated α-like tumors. In a second step using gene expression we found three distinct ADM subtypes: one with increased hypoxia signaling, one enriched in immune components, and the last group with no specific signature. Off note, the small size of ADM immunosuppressive PanNETs was not reflected in DFS, as they were equally prone to relapse as other ADM subtypes. Despite sharing loss in key driver genes, the four subtypes have very different expression profiles, suggesting distinct mechanisms driving progression, beyond the DAXX/ATRX loss of function.

We show that ADM PanNETs have hypomethylated subtelomeric DNA. One of the main functions of DAXX and ATRX is maintenance of telomeric structure [42]. DNA hypomethylation at subtelomeric regions is known to enhance sister chromatid exchange favoring homologous recombination, a mechanism on which depends ALT activation [43]. Subtelomeric hypomethylation has already been described in ALT positive cells in vitro [43] and in ALT positive tumors, such as glioblastoma and astrocytoma [44, 45]. Similarly, ATRX and H3F3A mutant glioblastoma showed hypomethylation of subtelomeric regions [44]. Analogous to subtelomeric regions, normal human centromeres and pericentromeres are overall hypermethylated. Loss of methylation at centromeric and pericentromeric regions might impair chromosomal segregation during cell division, enhancing chromosomal instability [46, 47].

Our data suggests that reduced methylation of sub-telomeric and peri-centromeric regions in ADM PanNETs may induce ALT activation and enhance genomic instability, linking these features with DAXX/ATRX loss (Fig. 2B and C). This is further illustrated by the progression from an α-like primary tumor to an ADM metastasis with activated ALT that we observed in one patient (Fig. 3A and B). Of note, ALT is known prognostic factor in PanNETs [13, 48–50]. ALT activation is associated with higher risk of relapse, grade, and size in both, functioning and NF-PanNETs [48–50]. In accordance, ADM PanNETs in the present study showed higher grade, increased ALT frequency, and elevated CNA burden compared to α-like tumors, supporting a link between hypomethylation, ALT activation, and chromosomal instability. Interestingly, previous studies revealed that patients with metastatic disease and ALT showed longer survival, while the opposite was true for non-metastatic tumors, suggesting a possible dual role in later PanNET progression [12, 51].While its association with DAXX/ATRX loss of function was already established previously, to our knowledge this is the first report correlating hypomethylation of telomeric and pericentromeric regions with ALT activation and CNA in PanNETs [48, 50], thus revealing possible driving mechanisms.

Further analysis of DNA methylation profiles of ADM PanNETs revealed a subtype characterized by hypoxia signalling and enriched in samples with homogenous MCT4 expression. This expression pattern of MCT4, not linked to local vascularization (Fig. 5B and Supplementary Fig. 5) suggests an oxygen-independent pseudo-hypoxic phenotype, possibly driven by epigenetic alterations (Fig. 1C) [52]. Indeed, promoter regions of many over-expressed genes related to hypoxia, glycolysis, and lactate metabolism, are hypomethylated in ADM hypoxic PanNET. Additional epigenetically regulated genes include also factors associated with epithelial-to-mesenchymal transition, a process that may be facilitated by (pseudo-)hypoxia [53].

Of the other two transcriptional ADM subclusters, ADM NST PanNETs showed different metabolic changes, including activation of oxidative phosphorylation, adipogenesis, and fatty acid metabolism (Fig. 4E). The ADM immunosuppressive subgroup is highly enriched in immune components, including allograft rejection, interferon γ and inflammatory response, or IL2 and IL6 signaling (Fig. 4E). For both groups expression changes could not be linked to epigenetic drivers, as we identified only 3 and no differentially up-regulated genes associated with promoter methylation, respectively.

Young and colleagues identified for the first time a metastasis-like primary (MLP1) PanNET subtype, which was associated with hypoxia, immune changes, and overall worse prognosis [15]. In our data, signature derived from MLP1 marker genes reached the highest score in both ADM hypoxic and ADM immunosuppressive PanNETs (Fig. 4F), highlighting similarities. A more recent proteogenomic study described two subtypes of PanNETs, C1 and C2, enriched in patients with mutations in *MEN1*, *DAXX*, and *ATRX* [54]. Our ADM-NST most closely resemble the C2 group, sharing features such as activation of fatty acid metabolism and a likely α-cell origin, whereas ADM-immunosuppressive are related to the C1 group, which is similarly characterized by an immunosuppressive phenotype. The authors also described a hypoxic subtype C4, primarily identified by an enrichment of mutation in *VHL*, and mutation in *MEN1*,* ATRX* and *DAXX*. These *VHL* mutated tumors showed immunohistochemistry staining independent from vessel density similarly to our hypoxic group [55]. Indeed, proposed markers of hypoxic PanNETs, namely P4HA1, PFKP, and SYNPO, were up regulated in the ADM hypoxic tumors described in this study (Supplementary Table 3) [54]. Additionally, Gucer and colleagues reported a case of inhibin-positive case of NET with Von Hippel-Lindau (VHL) syndrome. Evidence suggest that inhibin can be induced by HIF-1α, underlining its role in (pseudo-)hypoxic conditions [56]. In accordance, mRNA expression of inhibin subunit α (INHA) was up-regulated in ADM hypoxic PanNETs (Supplementary Fig. 6).

While ADM tumors have a higher risk of relapse compared to α-like PanNETs, their subtypes show similar prognosis (Supplementary Fig. 7) [2]. Interestingly, the ADM immunosuppressive comprise mainly tumors smaller than 2.5 cm (median = 2.4 cm), despite having invasive features like the larger PanNETs, indicating that molecular profiles can help to better stratify patients. Indeed, we have already described a high grade micro-PanNET [57].

Although proteogenomic profiling suggests that distinct PanNET subtypes may respond differently to targeted therapies, DNA methylation profiles were not considered in that study [54]. This limitation may hinder the discrimination of small, indolent tumors and lead to insufficient stratification of patients who could benefit from less aggressive treatment strategies. Whether the subtypes identified here would respond differently to specific therapeutic approaches remains to be elucidated.

In conclusion we could dissect three subtypes of PanNET with mutations in *MEN1*, *DAXX* and *ATRX* which seem to follow different mechanisms of progression including two different metabolic phenotypes and a phenotype of immune evasion. Possibly, these findings may open the way to novel, more targeted treatments.

## Supplementary Information

Below is the link to the electronic supplementary material.

Supplementary Material 1 (DOCX 1.63 MB)

Supplementary Material 2 (DOCX 807 KB)

## Data Availability

The authors declare that all data supporting the findings of this study are available within the article and its Supplementary data and figuress. The new DNA methylation datasets generated during the current study (University of Bern, UB and UB_2020 cohort) is available in the ArrayExpress repository (EMBL-EBI, https://www.ebi.ac.uk/arrayexpress/, accession number: E-MTAB-11962 and E-MTAB-11965. Data from the UB_2025 cohort is available from European Genome-phenome Archive (EGA, accession number: EGAS00001008272). The previously published DNA methylation datasets analysed during the current study are available in the ArrayExpress repository (EMBL-EBI, https://www.ebi.ac.uk/arrayexpress/, accession number: E-MTAB-7924), International Cancer Genome Consortium (ICGC) repository (ICGC, https://icgc.org/, projects: PAEN-AU and PAEN-IT) and in the GEO repository (Gene Expression Omnibus, http://www.ncbi.nlm.nih.gov/geo/, accession number: GSE117852). The new RNAseq datasets generated during the current study (UB_2025 cohort) are available from European Genome-phenome Archive (EGA, accession number: EGAS00001008272). The previously published RNAseq datasets for PanNET analysed during the current study are available in the GEO repository (Gene Expression Omnibus (GEO), http://www.ncbi.nlm.nih.gov/geo/, accession number: GSE118014) and in the ICGC repository (ICGC, https://icgc.org/, projects: PAEN-AU and PAEN-IT).

## Abbreviations

ADM: ATRX/DAXX and MEN1 loss
ALT: Alternative lengthening of telomeres
CNA: Copy number variations
ICGC: International Cancer Genome Consortium
MAD: Median absolute deviation
MLP1: Metastasis like primary 1
NF: Non–functioning
NST: No special type
PanNET: Pancreatic Neuroendocrine Tumors
UB: University of Bern
UCL: University College London
