# Correction to: Molecular Subtypes of Pancreatic Neuroendocrine Tumors Mutated in *MEN1/DAXX/ATRX* Explain Biological Variability

**DOI:** 10.1007/s12022-025-09892-x

**Published:** 2025-11-26

**Authors:** Simona Avanthay, Annunziata Di Domenico, Philipp Kirchner, Konstantin Bräutigam, Aziz Chouchane, Renaud Maire, Christina Thirlwell, Corina Kim-Fuchs, Aurel Perren, Ilaria Marinoni

**Affiliations:** 1 Institute of Tissue Medicine and Pathology, University of Bern, 3008 Bern, Switzerland; 2 Graduate School for Cellular and Biomedical Sciences, University of Bern, Bern, Switzerland; 3 Bristol Medical School, University of Bristol, Bristol, England UK; 4 Department of Visceral Surgery and Medicine, University Hospital Bern, University of Bern, Bern, Switzerland

**Correction to: Endocrine Pathology**

10.1007/s12022-025-09889-6

The original published version of this article unfortunately contained an error.

Figure 1 was presented as:
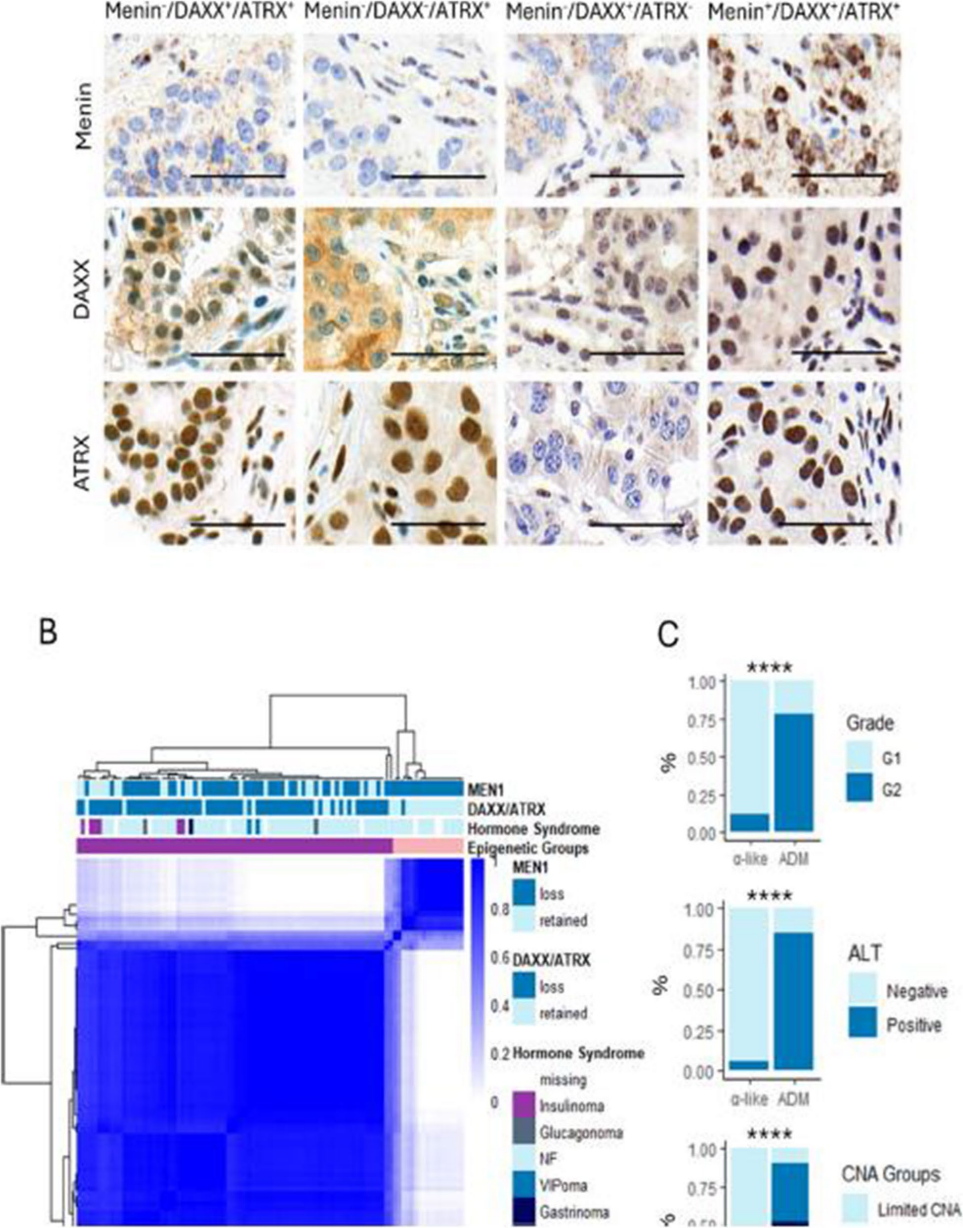


This should be corrected to:
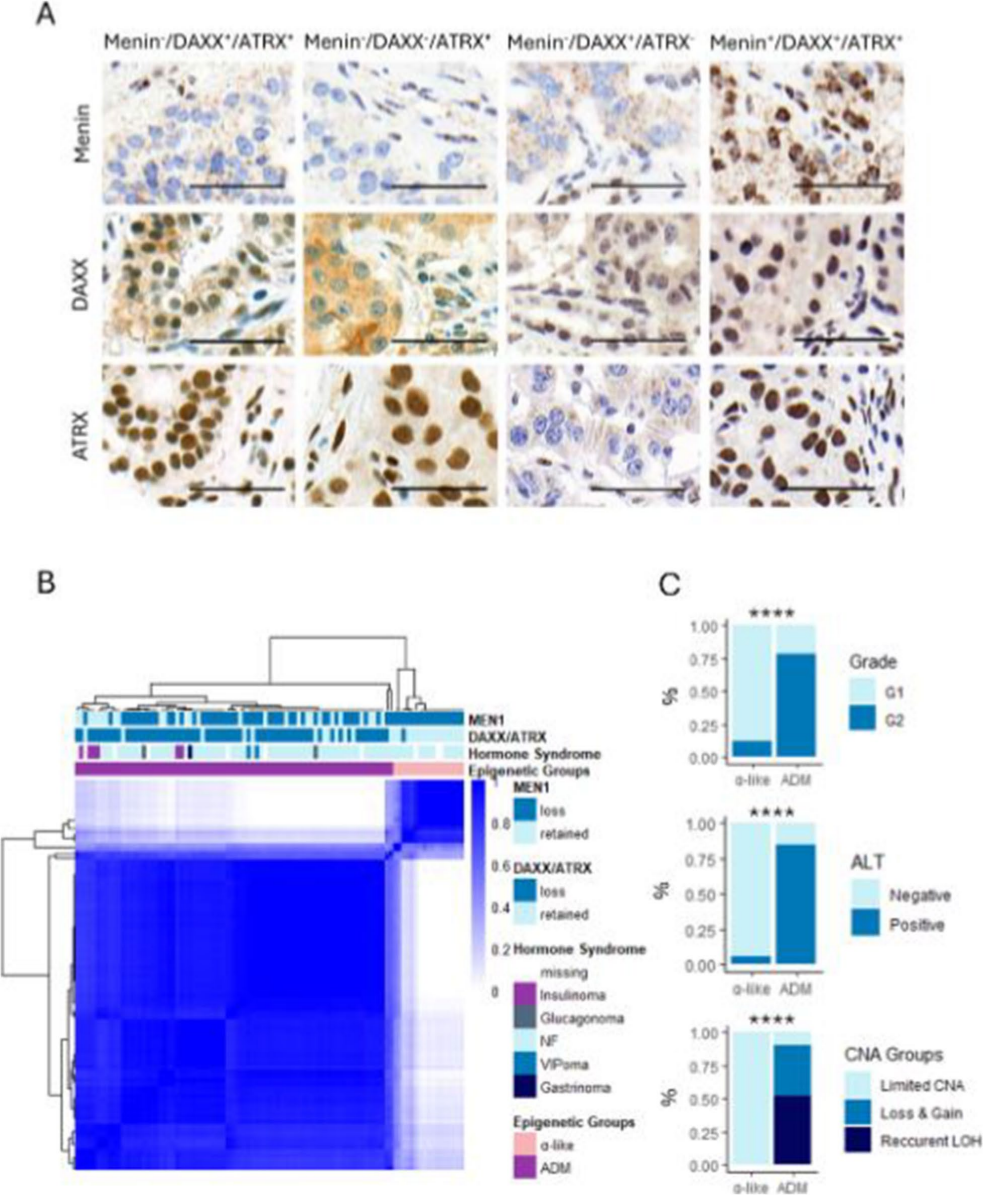


The original article has been corrected.

