# Supplementary material for: Molecular Subtypes of Pancreatic Neuroendocrine Tumors Mutated in MEN1/DAXX/ATRX Explain Biological Variability
Source: Endocr Pathol. 2025 Nov 10;36(1):44. doi: 10.1007/s12022-025-09889-6 (PMC12602606; doi:10.1007/s12022-025-09889-6)
Supplement: Supplementary file 1 — Supplementary Material 1 (DOCX 1.63 MB) [file 12022_2025_9889_MOESM1_ESM.docx]

# Supplementary Figures


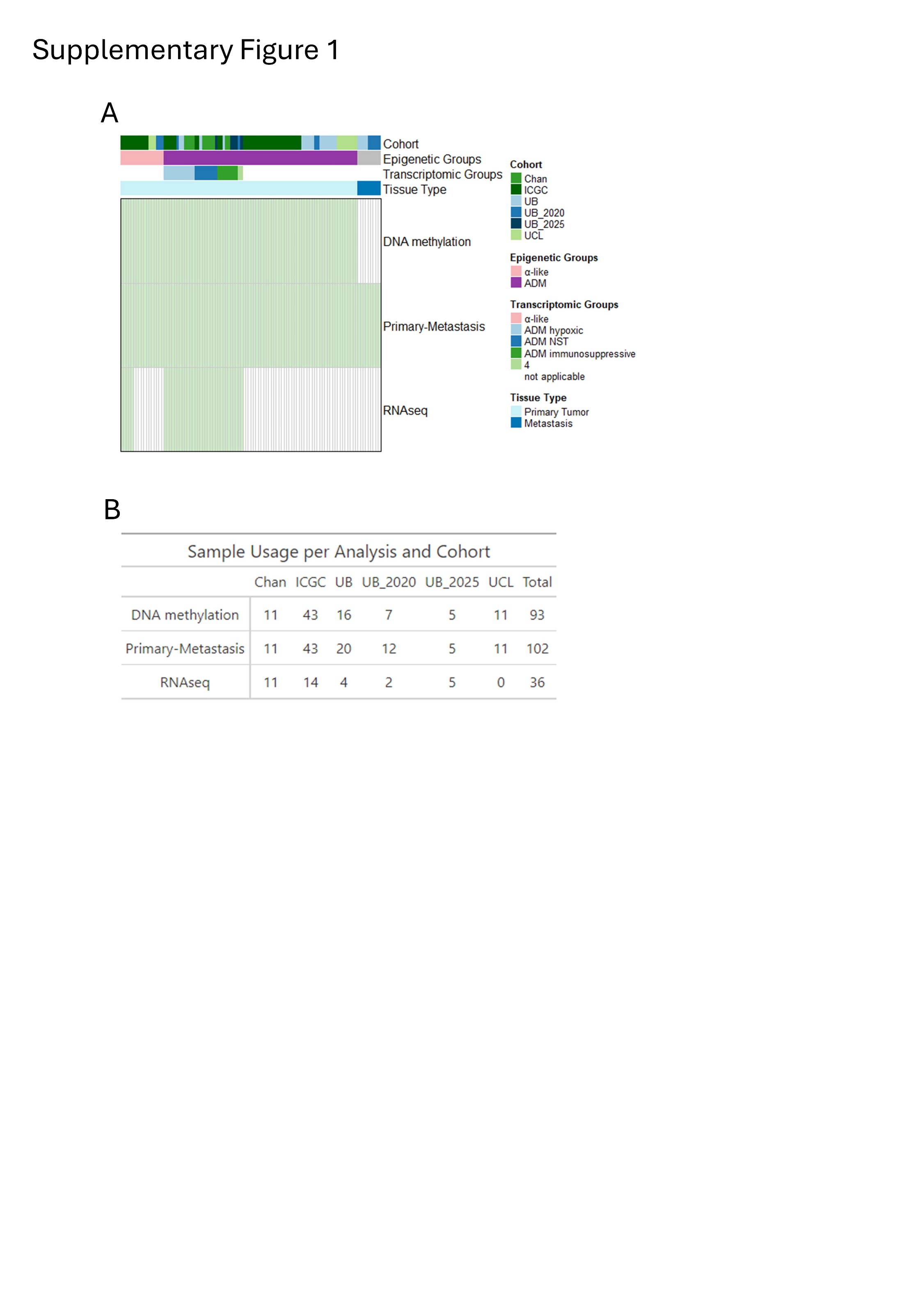


^1^Chan CS et al. (2018) ATRX, DAXX or MEN1 mutant pancreatic neuroendocrine tumors are a distinct alpha-cell signature subgroup. Nat Commun 9:. <https://doi.org/10.1038/s41467-018-06498-2>;

^2^Scarpa A et al (2017) Whole-genome landscape of pancreatic neuroendocrine tumours. Nature 543:65–71 https://doi.org/10.1038/nature21063;

^3^Di Domenico A, Pipinikas CP, Maire RS, et al (2020) Epigenetic landscape of pancreatic neuroendocrine tumours reveals distinct cells of origin and means of tumour progression. Commun Biol 3: <https://doi.org/10.1038/s42003-020-01479-y>

1B

1A

3

3c

2c

1c

## Supplementary Fig.1:

**A.** Visualization of samples used for each analysis with respective epigenetic or transcriptomic groups, original publication, and tissue type as indicated according to the color legend above. Every column represents a sample. Rows indicate specific analyses. Green and white colors in the main matrix indicate whether the sample was included in the specific analysis (green-yes, white-no). **B.** Number of samples from each cohort used for respective analysis. Chan: Chan *et al*. [21], ICGC (International Cancer Genome Consortium): Scarpa *et al.* [5], UB (University of Bern) and UCL (University College London): Di Domenico *et al*. [19], UB_2020 and UB_2025 are additional samples with previously unpublished data from University of Bern.


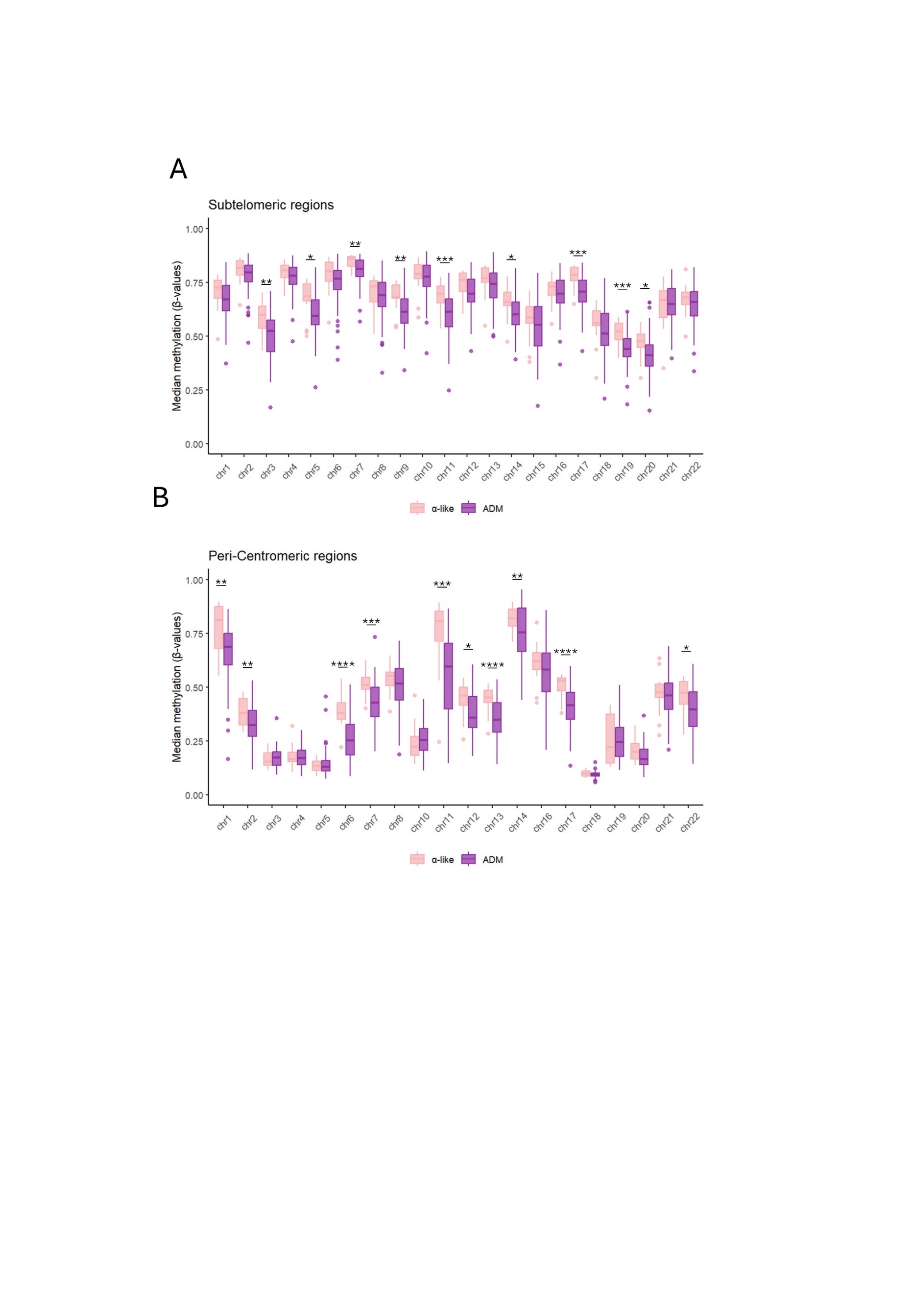


2A

2B

## Supplementary Fig.2:

Median methylation β-values of subtelomeric (**A**) and (peri-)centromeric (**B**) regions for each chromosome. PanNET subgroups colored as depicted in the color legend. *p-value <0.05, **p-value<0.01, ***p-value<0.001, ****p-value<0.0001


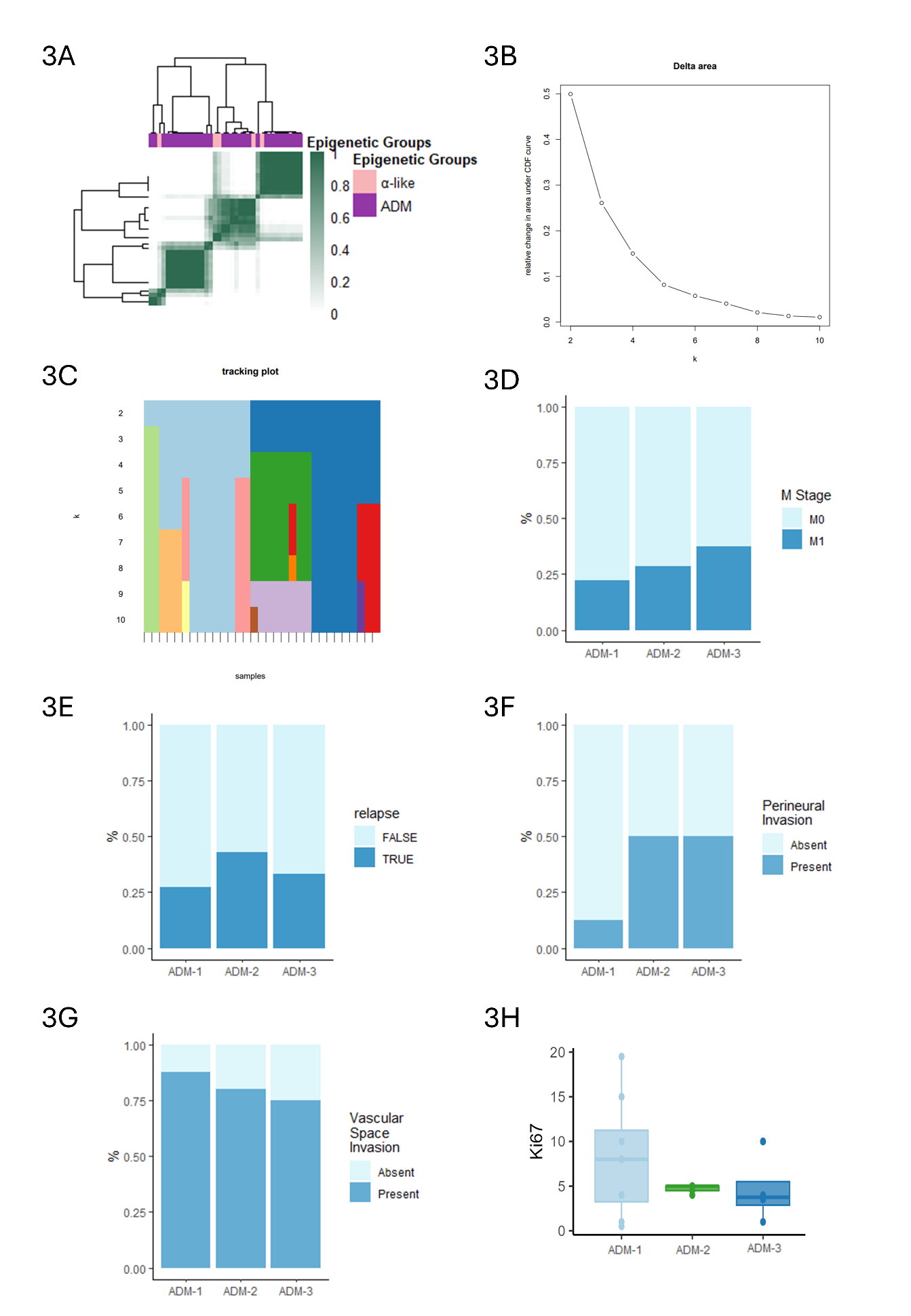


## Supplementary Fig.3:


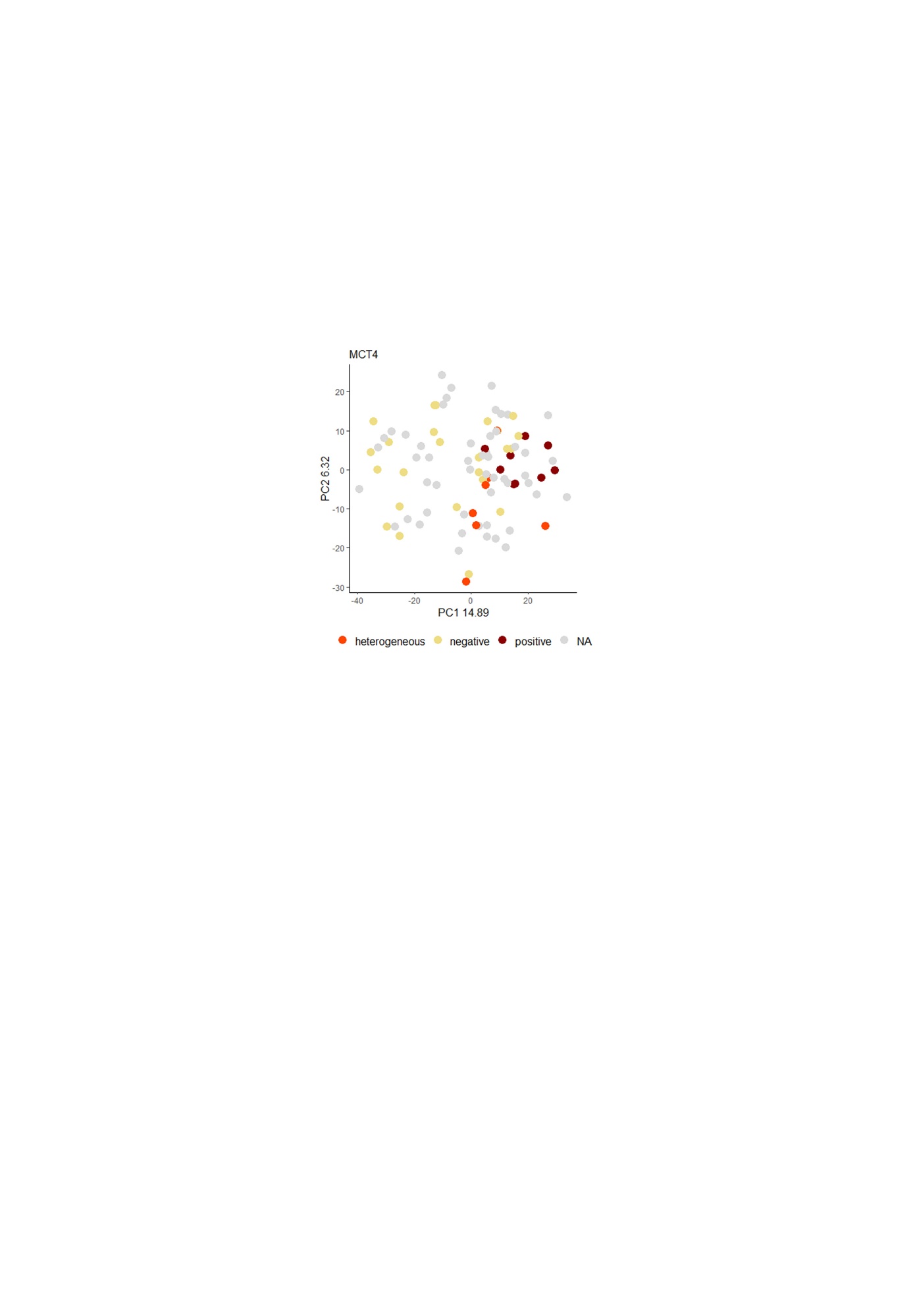
**A.** Consensus clustering of the 36 PanNETs according to the most variably expressed genes (calculated by MAD). Consensus cluster correlation is indicated according to the white to green scale with darker colors representing higher correlation. Each column represents one sample. Epigenetic groups are reported according to the color key. Cluster stability was reached for *k*=4. **B**. plot of cumulative distribution functions (CDF) for the consensus matrices of ADM PanNETs for each *k.* **C***.* Tracking plot representing samples distribution for k=2 to k=10. Colors for k=4 represent respective ADM subtypes (light blue for ADM-1, dark blue for ADM-2, and dark green for ADM-3). **D**. Bar plots depicting percentage of samples in given ADM subtype with specific M stage or relapse.

4

## Supplementary Fig.4:

PCA on top 2000 most variably methylated probes (according to MAD). Each dot represents a sample. MCT4 protein expression profile as identified by immunohistochemistry is reported according to the color key.

5


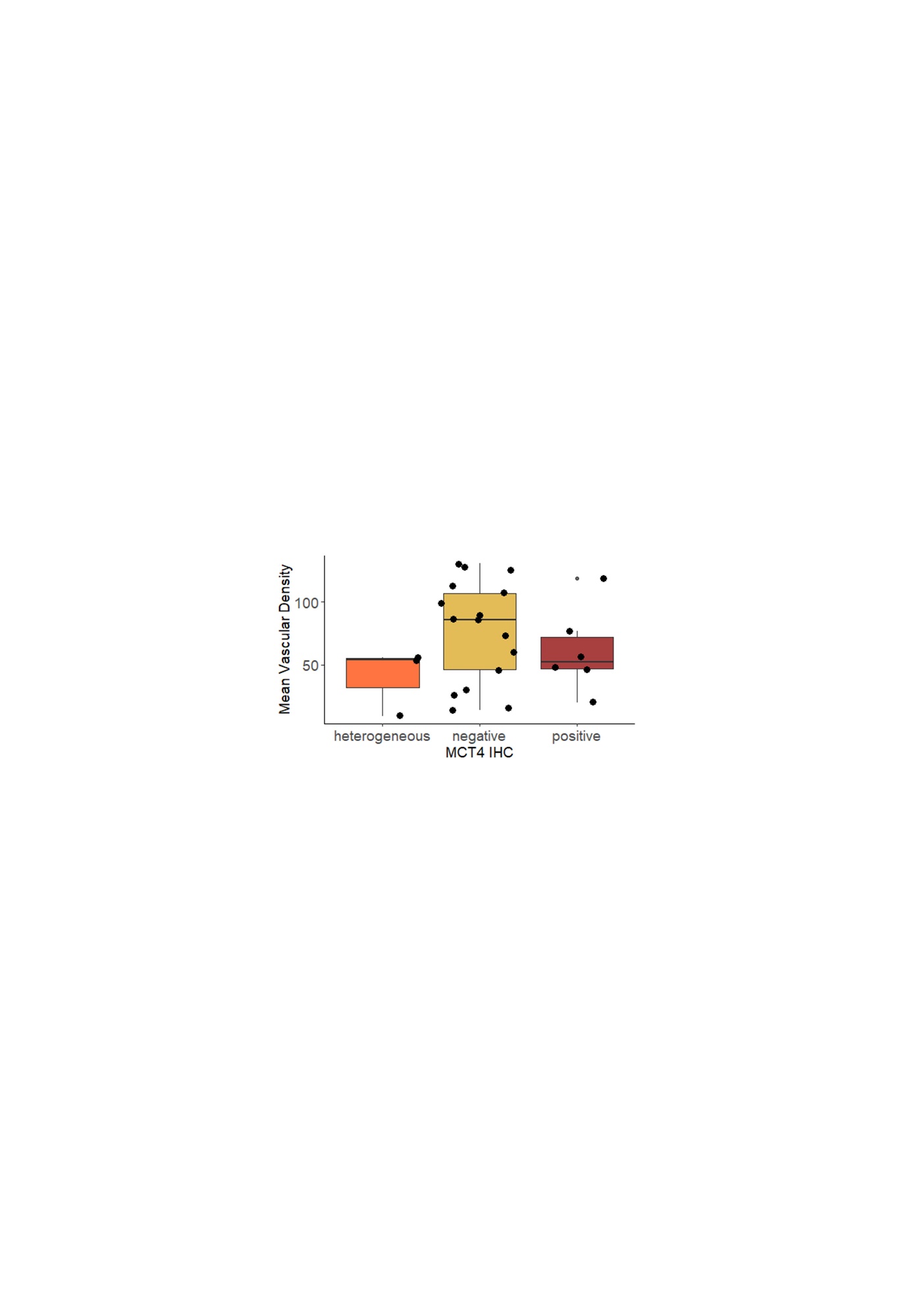


5

## Supplementary Fig.5:

Mean vascular density (y axis) in samples with different MCT4 protein expression profile as assessed by immunohistochemistry.


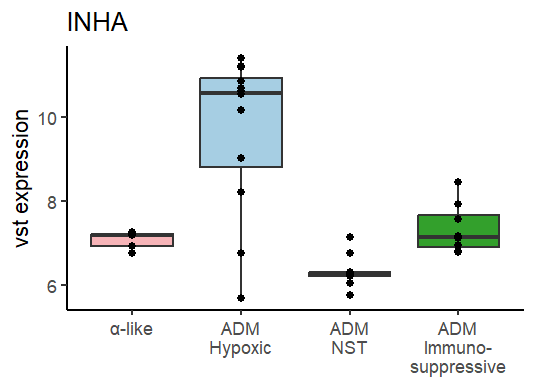


6

## Supplementary Fig.6:

VST-normalized gene expression of INHA is the highest in ADM hypoxic tumors.

##
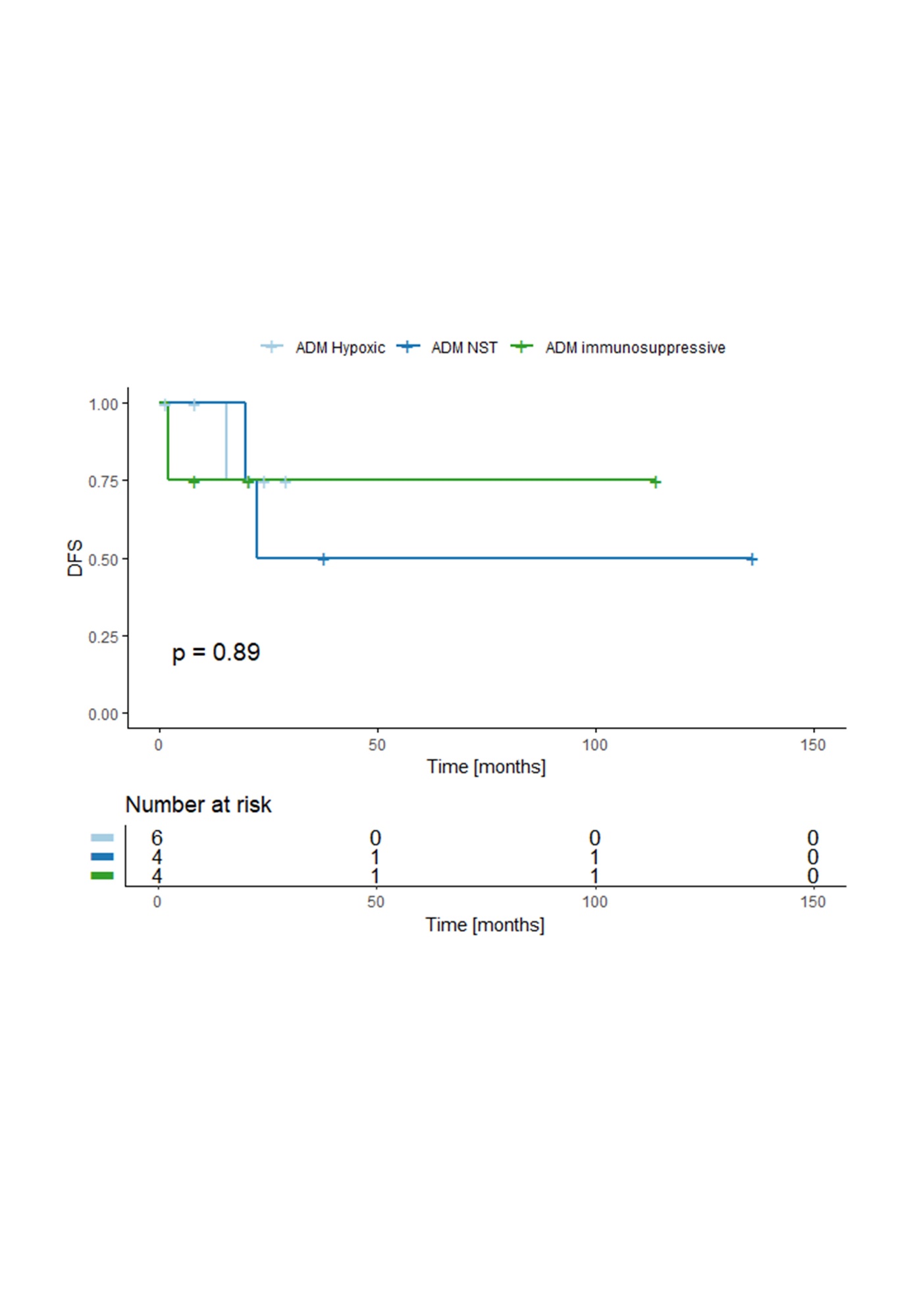
Supplementary Fig.7:

7

Kaplan-Meier analysis of disease-free survival with the p value of a log-rank test. The table shows the number at risk for 0-, 50-, 100- and 150-months disease free time respectively.
